# Gene signature predicting recurrence in oral squamous cell carcinoma is characterized by increased oxidative phosphorylation

**DOI:** 10.1002/1878-0261.13328

**Published:** 2022-11-23

**Authors:** Joo Kyung Noh, Seon Rang Woo, Moonkyoo Kong, Min Kyeong Lee, Jung Woo Lee, Young Chan Lee, Seong‐Gyu Ko, Young‐Gyu Eun

**Affiliations:** ^1^ Department of Biomedical Science and Technology, Graduate School Kyung Hee University Seoul Korea; ^2^ Department of Otolaryngology‐Head & Neck Surgery, Kyung Hee University School of Medicine Kyung Hee University Medical Center Seoul Korea; ^3^ Department of Radiation Oncology, Kyung Hee University School of Medicine Kyung Hee University Medical Center Seoul Korea; ^4^ Department of Oral and Maxillofacial Surgery, School of Dentistry Kyung Hee University Seoul Korea; ^5^ Department of Preventive Medicine, College of Korean Medicine Kyung Hee University Seoul Korea

**Keywords:** gene signature, MED30, mitochondrial membrane potential, oral squamous cell carcinoma, oxidative phosphorylation

## Abstract

Although numerous studies have used systemic approaches to identify prognostic predictors in oral squamous cell carcinoma (OSCC), the effectiveness of these approaches has not been assessed clinically. Further, the mechanism underlying malignant behaviors in OSCC is poorly characterized. This study aimed to develop and verify accurate prognostic predictors for OSCC patients and assess the associated biology. We identified an OSCC‐recurrence‐related gene signature (ORGS) using a Cox regression analysis. Functional enrichment analysis was used to identify enriched pathways and biological processes to reveal the underlying mechanism of OSCC malignant behavior. The ORGS successfully divided OSCC patients into low‐ and high‐risk groups with significantly different overall survivals. Pathway analysis revealed oxidative phosphorylation (OXPHOS) as a signaling pathway associated with the ORGS in OSCC. Interestingly, high OXPHOS status was strongly associated with poor overall survival in OSCC patients. Mediator complex subunit 30 (MED30) was a predicted upstream regulator of OXPHOS, and knockdown of MED30 reduced histone acetylation. We identified that the ORGS was strongly correlated with OXPHOS regulatory processes, suggesting OXPHOS as a key mechanism leading to poor prognosis in OSCC.

## Graphical abstract

Here, we define a gene signature from oral squamous cell carcinoma (OSCC) patients which could stratify patients into low and high‐risk groups with different overall survival. We highlight oxidative phosphorylation (OXPHOS) as a signaling pathway associated with this signature. Finally, we demonstrate that MED30‐induced OXPHOS gene transcription depolarizes mitochondrial membrane potential and inhibits ROS‐dependent apoptosis of cancer cells.

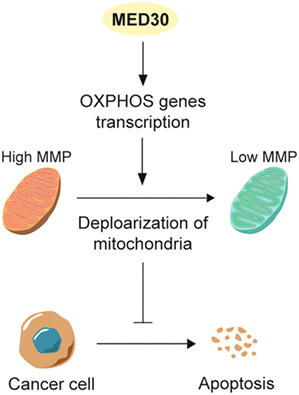

**Abbreviations**
AML: acute myeloid leukemia
CCA: cholangiocarcinoma
cDNA: complementary DNA
CSCs: cancer stem cells
ETC: electron transport chain
FC: fold change
FHCRC: Fred Hutchinson Cancer Research Center
GO: gene ontology
GSCs: glioblastoma stem cells
HNSCC: head and neck squamous cell carcinoma
HR: Hazard ratio
IPA: ingenuity pathway analysis
KEGG: Kyoto Encyclopedia of Genes And Genomes
KHU: Kyung Hee University
MED30: mediator complex subunit 30
MMP: mitochondrial membrane potential
NAC: *N*‐acetyl‐cysteine
ORGS: OSCC recurrence‐related gene signature
OSCC: oral squamous cell carcinoma
OXPHOS: oxidative phosphorylation
PBS: phosphate‐buffered saline
PCR: polymerase chain reaction
PGC‐1α: proliferator‐activated receptor gamma coactivator‐1 alpha
PI: propidium Iodide
qPCR: quantitative polymerase chain reaction
ROS: reactive oxygen species
SVM: support vector machine
TCGA: The Cancer Genome Atlas

## 1 Introduction

Oral squamous cell carcinoma (OSCC) is the most common type of oral cancer, accounting for over 90% of all oral cancers [1]. In 2020, there were an estimated 430 000 new OSCC diagnoses and approximately 200 000 related deaths worldwide [2]. Despite improvements in surgery and adjuvant radio‐ and chemotherapy, the prognosis of OSCC is still poor, with a median survival of 37.6 months and a 5‐year survival rate of 64.4% [3]. The causes of this poor prognosis include aggressive local invasion, metastasis, and recurrence. A fundamental goal of cancer research is to understand the mechanisms of tumor recurrence, thereby overcoming the failure of radiotherapy and chemotherapy in cancer. A systematic approach including genomics is key to developing more efficient prognostic and predictive factors for cancer.

Oxidative phosphorylation (OXPHOS) is a metabolic pathway that generates ATP. As electrons pass through the electron transport chain (ETC), protons are pumped from the mitochondrial matrix into the intermembrane space. When OXPHOS is active, these protons flow from the inner intermembrane space back into the mitochondrial matrix through the ETC complex, driving ATP synthesis [4]. Metabolic reprogramming is an emerging hallmark of cancer. Early studies on the energy metabolism of cancer cells revealed that glycolysis is increased in cancer cells compared to normal cells. Aerobic glycolysis is generally accepted as a metabolic hallmark of cancer. It is also accepted that cancer cells depend on aerobic glycolysis, in contrast to normal differentiated cells, which rely on OXPHOS [5].

However, recent studies have shown that OXPHOS is increased in some cancers, including breast cancer, lung cancer, and acute myeloid leukemia (AML) [6, 7, 8]. Moreover, OXPHOS is the preferred energy‐production process in cancer stem cells (CSCs) [9]. Cholangiocarcinoma (CCA) CSCs adopt a more efficient respiratory phenotype and depend on OXPHOS, overexpressing peroxisome proliferator‐activated receptor gamma coactivator (PGC)‐1α, a master regulator of mitochondrial biogenesis [10]. Patients with CCA and high OXPHOS activity have a shorter overall survival and time to recurrence than those with low OXPHOS. Glioblastoma stem cells (GSCs) also highly rely on OXPHOS [11]. Induction of OXPHOS in GSCs leads to the failure of glioblastoma therapies. These studies suggest that targeting OXPHOS therapeutically may suppress cancer malignancy.

Therefore, this study was designed to develop and validate a novel and robust gene signature that can predict prognosis in OSCC. In addition, it aimed to reveal the malignant and progressive mechanisms leading to treatment failure in OSCC patients to identify therapeutic strategies for OSCC.

## 2 Materials and methods

### 2.1 Patients and cohorts

A total of 20 530 gene expression data plus clinical information for 520 Head and Neck Squamous Cell Carcinoma (HNSCC), 43 matched non‐tumor samples and 1 unmatched non‐tumor samples were downloaded from The Cancer Genome Atlas (TCGA, https://gdc.cancer.gov/) database (assessed October 2020). The sequencing profile data of patients with OSCC were obtained from TCGA. TCGA cohort data were downloaded from the UCSC Cancer Genomics Browser (http://xena.ucsc.edu/) and used as the training dataset. Two independent datasets, a dataset from the Fred Hutchinson Cancer Research Center (FHCRC; GSE41613) [12] and a dataset from our institution (Kyung Hee University [KHU]), were used for independent validation. The FHCRC cohort data were downloaded from the National Center for Biotechnology Information Gene Expression Omnibus database (https://www.ncbi.nlm.nih.gov/geo/). The FHCRC cohort obtained 54 613 microarray gene expression data and clinical data of 97 HPV‐negative HNSCC patients. The KHU cohort was obtained from the Department of Otolaryngology‐Head and Neck Surgery, School of Medicine, Kyung Hee University. The experiments were undertaken with the understanding and written consent of each subject. The study methodologies were approved by our institutional review board (IRB: 2018‐05‐046‐12) and were conducted according to the principles of the Declaration of Helsinki. Table 1 shows the clinical and pathological characteristics of patients in each cohort. The TCGA, FHCRC, and KHU cohorts had 315, 87, and 45 patients with OSCC, respectively.

**Table 1:** Oral cavity cancer patient's information of TCGA, FHCRC and KHU cohort.

	TCGA	FHCRC.Seattle	KHU
Number of patients	315	97	45
Gender			
Male	213	66	32
Female	102	31	13
Age			
≥ 60 years old	178	47	26
< 60 years old	136	50	19
Primary tumor			
T1	31		11
T2	97		9
T3	59		0
T4	110		25
Regional lymph node			
N0	119		30
N1	47		5
N2	101		10
N3	3		0
Stage			
I	20	41	8
II	54		5
III	56	56	1
IV	162		18
HPV status			
Positive	15	0	
Negative	300	97	
Smoking			
Yes	219		25
No	86		20
Alcohol			
Yes	203		
No	105		

### 2.2 Tissue collection

Tissue samples from 45 OSCC patients were collected and assessed using RNA‐seq. This study was conducted at Kyung Hee University Medical Center from Jan 2011 to Jan 2019. Tissue samples (surgical specimens or biopsies) from head and neck squamous cell carcinoma (HNSCC) patients were collected in a tube containing the stabilizing reagent TRIzol (Invitrogen, Waltham, MA, USA). The tube was then quickly placed in liquid nitrogen. Samples were transported to the patient specimen storage facility within 1 h, snap‐frozen, and stored at −80 °C.

### 2.3 RNA‐seq

RNA was extracted using TRIzol Reagent as previously described [13]. Briefly, the tissues were homogenized in TRIzol reagent. The homogenate was mixed with chloroform and centrifuged to obtain the top aqueous phase, interphase, and bottom organic phase. RNA was precipitated from the aqueous phase by adding isopropanol, washed, and dissolved in DEPC‐treated water.

The total RNA quality and quantity were verified spectrophotometrically (NanoDrop spectrometer; Thermo Scientific, Waltham, MA, USA) and electrophoretically (Bioanalyzer 2100; Agilent Technologies, Santa Clara, CA, USA). A TruSeq RNA library preparation kit (Illumina, San Diego, CA, USA) was used to construct Illumina‐compatible libraries, according to the manufacturer's instructions. Briefly, messenger RNA purified from total RNA using polyA selection was chemically fragmented and converted into single‐stranded complementary DNA (cDNA) using random hexamer priming. Double‐stranded (ds) cDNA was generated for TruSeq library construction. Short ds‐cDNA fragments were ligated to sequencing adapters, and suitable fragments were separated by agarose gel electrophoresis. TruSeq RNA libraries constructed by polymerase chain reaction (PCR) amplification were quantified by quantitative PCR (qPCR) according to the qPCR Quantification Protocol Guide, and their quality was assessed electrophoretically using a Bioanalyzer 2100. Sequencing was performed using the HiSeq™ 200 platform (Illumina).

### 2.4 Development of the gene signature

Using each gene as one variable, a Cox proportional hazards model was used to determine which genes affected the survival rate. The cutoff *P*‐value was 0.005, and 210 genes were selected as the OSCC recurrence‐related gene signature (ORGS). Among the 210 genes, 123 genes with hazard ratios (HRs) > 1 were classified as high‐risk genes, and 87 genes with HRs < 1 were classified as low‐risk genes. Table S1 shows the genes included in the ORGS and their HRs and *P*‐values. We used data from 315 OSCC patients in TCGA to confirm that the ORGS could predict RFS. Hierarchical clustering was performed using cluster 3.0 [14], and a heatmap was plotted using java treeview [15]. Survival curves were generated and analyzed using the Kaplan–Meier method with the log‐rank test. Cox proportional hazard model and survival analyses were performed using r (http://www.r‐project.org), and *P* < 0.05 was considered statistically significant.

### 2.5 Validation of the gene signature in independent cohorts

We validated the gene expression signature by classifying patients with HNSCC from independent cohorts using a support vector machine (SVM) [16]. Gene expression data from TCGA (training set) were grafted into the SVM algorithm to generate discriminators. The SVM predictor is a linear function of log‐ratios or log‐intensities that allows for better data separation based on the penalty cost of the misclassified specimen number. Validation was conducted in the independent FHCRC and KHU cohorts. Predictive model construction and validation were performed using the BRB‐array tools (https://brb.nci.nih.gov/BRB‐ArrayTools/).

### 2.6 Gene ontology and pathway enrichment analysis

To analyze the functions and pathways associated with the ORGS, gene ontology (GO) [17] and Kyoto Encyclopedia of Genes And Genomes (KEGG) [18] pathway enrichment analyses were performed. The term *P*‐value was set at 0.05, and terms including at least three genes were retrieved. The potential functions of target genes in biological processes were analyzed using GO terms. KEGG is a database used for the systematic analysis of gene functions and utilities in biological systems using molecular‐level information. We obtained information on both the GO terms and KEGG pathways using the cluego plug‐in (2.5.6) [19] in cytoscape version 3.7.0 [20].

### 2.7 Ingenuity pathway analysis upstream regulator analysis

Co‐expressed genes participating in the same biological process or disease may be regulated by the same or similar regulators, especially transcription factors. To explain the biological activities of OXPHOS gene, a list of OXPHOS genes, containing gene identifiers and the corresponding *P*‐value in the Cox proportional hazard model, was uploaded into the ingenuity pathway analysis (ipa) software. The ‘core analysis’ function was used to identify upstream transcriptional regulators. We examined the upstream transcriptional regulators of OXPHOS genes with a *P*‐value of overlap < 0.01.

### 2.8 Cell culture

The human OSCC cell lines HSC3 and HSC4 were purchased from the Japanese Collection of Research Bioresources Cell Bank. The cells were cultured in Roswell Park Memorial Institute‐1640 medium containing 10% heat‐inactivated fetal bovine serum (Hyclone, Logan, UT, USA) and 1% penicillin–streptomycin (Corning Inc., Cornin, NY, USA). All cell lines were maintained at 37 °C in a humidified incubator containing 5% CO_2_.

### 2.9 Transfection

Cells were transfected with negative control siRNA (Bioneer, Daejeon, Republic of Korea) or siMED30 (Bioneer, 90390‐1 and 90390‐2) using Lipofectamine RNAiMAX Transfection Reagent (Thermo Fisher Scientific, Waltham, MA, USA), according to the manufacturer's instructions.

### 2.10 Quantitative real‐time PCR

Cells were seeded in 6‐well plates and transfected with siRNA. Total RNA was extracted using TRIzol‐LS reagent (GeneAll, Seoul, Republic of Korea), according to the manufacturer's protocol. We used a NanoDrop spectrophotometer to assess RNA concentration and quality. Extracted RNA was reverse‐transcribed into cDNA using PrimeScript RT Master Mix (Takara Bio, RR036A, Kysatsu, Japan). Quantitative real‐time PCR (qRT‐PCR) was performed using TB Green Premix Ex Taq II (Takara Bio, RR820A).

PCR was performed under the following conditions: initial denaturation at 95 °C for 30 s followed by 40 cycles of denaturation at 95 °C for 5 s and annealing at 60 °C for 30 s. The PCR reaction was evaluated using melting curve analysis. Each sample was amplified in triplicate, and the data were analyzed by relative quantitation using the ΔΔCt method and normalized to β‐actin. The primer sequences are listed in Table S2. The mean of three independent biological replicates was calculated. The fold change (FC) was calculated as the mean expression in cells transfected with siMED30 divided by the mean expression in cells transfected with siCON. The FC represents changes in transcript levels in cells transfected with siMED30 compared to those in control cells. Statistical analysis of the difference between siCON and siMED30 cells was performed using a two‐tailed *t*‐test (graphpad prism, GraphPad, San Diego, CA, USA). Statistical significance was set at *P* < 0.05.

### 2.11 Western blotting

Cells were homogenized in RIPA buffer (1% Triton X‐100, 1% sodium deoxycholate, 0.1% SDS, 150 mm NaCl, 50 mm Tris–HCl [pH 7.5], and 2 mm EDTA [pH 8.0]; Biosesang, Seongnam, Republic of Korea). Total protein was quantified using the BCA Protein Quantification Kit (Thermo Fisher Scientific) according to the manufacturer's protocol. Equal amounts of protein and loading dye (5× SDS/PAGE loading buffer; iNtRON Biotechnology, Seongnam, Republic of Korea) were added per lane and resolved using an SDS‐polyacrylamide gel. After electrophoresis, samples were transferred to polyvinylidene difluoride membranes (Millipore, Burlington, MA, USA), which were then blocked for 1 h in 5% skim milk and TBS with 0.1% Tween‐20 and incubated overnight at 4 °C with primary antibodies. The following antibodies were used: anti‐MED30 (sc‐393289, mouse monoclonal antibody, Santa Cruz Biotechnology, Dallas, TX, USA), anti‐actin (sc‐47778, mouse monoclonal antibody, Santa Cruz Biotechnology), anti‐histone H2A (#12349, Cell Signaling Technology, Danvers, MA, USA), anti‐acetyl‐histone H2A (#2576, Cell Signaling Technology), anti‐histone H2B (#12364, Cell Signaling Technology), and anti‐acetyl‐histone H2B (#9649, Cell Signaling Technology). Membranes were incubated with the corresponding secondary antibodies (Cell Signaling Technology) for 1 h at room temperature, followed by visualization using a chemiluminescence detection kit (RPN2232; GE Healthcare, Chicago, IL, USA). The quantification of band density was measured using image j (madison, wi, usa and standardized with the band density of β‐actin.

### 2.12 Mito‐id

Mitochondrial membrane potential was assessed using a MITO‐ID Membrane Potential Detection Kit (ENZ‐51018, Enzo Life Sciences Inc., Farmingdale, NY, USA) in accordance with the manufacturer's instructions. Cells were seeded onto a glass slide. After transfection with siMED30 or siCON, the cells were washed with 1× Assay Solution, and 5 μL of MITO‐ID MP detection reagent and 2 μL of necrosis detection reagent were added with 500 μL of assay solution. Cells were protected from light and incubated for 15 min at room temperature. The slides were then mounted using Vectashield mounting medium (Vector Laboratories, Newark, CA, USA). Fluorescent images were obtained using a confocal microscope (Zeiss LSM710, Jena, German).

Mitochondrial membrane potential (MMP) was measured using a MITO‐ID Membrane Potential Detection Kit (ENZ‐51018, Enzo Life Sciences Inc.) following the manufacturer's protocol. After incubation, cells were collected and washed with phosphate‐buffered saline (PBS). The samples were preincubated in 500 μL of assay solution containing 5 μL of MITO‐ID MP detection reagent and 2 μL of necrosis detection reagent. The samples were protected from light, incubated for 15 min at room temperature, and analyzed by flow cytometry (BD Pharmingen, BD Biosciences, Franklin Lakes, NJ, USA). The FL‐1 channel detected depolarized mitochondria, indicated by green fluorescence, and the FL‐2 channel detected orange fluorescence from energized mitochondria. Statistical analysis (Kolmogorov–Smirnov test) was used to assess differences between siCON and siMED30 samples; *P* < 0.05 was considered to be significant.

### 2.13 MitoSOX red assay

The generation of reactive oxygen species (ROS) in cells was detected using the MitoSOX Red Mitochondrial Superoxide Indicator (Invitrogen). After transfection with siMED30 or siCON, cells were washed with phosphate‐buffered saline (PBS) and incubated with 5 μmol·L^−1^ MitoSOX Red for 10 min at 37 °C in the dark. The cells were gently washed three times with warm PBS. Stained cells were mounted using Vectashield mounting medium (Vector Laboratories, Burlingame, CA, USA) and imaged using a confocal microscope (Zeiss LSM710).

### 2.14 Apoptosis analysis

Apoptosis was analyzed using an annexin V‐FITC apoptosis detection kit (Biobud, Seoul, Republic of Korea). Cells were transfected with siMED30 or siCON. After 24 h siMED30 transfection, 2 mm of *N*‐Acetyl Cysteine (NAC, Sigma, St.Louis, MO, USA) was treated in the HSC3 and HSC4 cells. After 48 h incubation, cells were trypsinized (TrypLE Express, Gibco, Thermo Fisher Scientific) and collected in conical tubes. The cells were centrifuged at 1000 × ***g*** for 5 min at room temperature and then washed with cold PBS. The cells were then centrifuged again at 1000 × ***g*** for 5 min. Then, 500 μL 1× binding buffer and 1.25 μL annexin V‐FITC was added to the cells, and cells were incubated for 15 min at RT in the dark. Next, 10 μL propidium iodide (PI) was added to the cells, and the cells were covered with aluminum foil. Flow cytometry (BD Pharmingen) was used to assess the proportion of annexin V‐positive cells. Statistical analysis (two‐tailed *t*‐test) was performed to assess differences between siCON and siMED30‐transfected cells, and *P* < 0.001 was defined as statistically significant.

### 2.15 Transwell invasion assay

The invasion assays were performed using a Transwell chamber (3422, Corning Inc.). For invasion assay, the chamber was coated with Matrigel (354234, Corning Inc.). The transfected cells were seeded into the upper chamber with RPMI with 0.1% FBS (3 × 10^4^ cells), and the bottom of the chamber contained the RPMI with 10% FBS. After the cells invaded for 48 h, they were fixed and stained with Hematoxylin (YD Diagnostics, Yongin, Republic of Korea & Eosin Y (Sigma). The number of invaded cells were quantified by counting the number of cells from six random fields at ×100 magnification.

### 2.16 Acquisition of differentially expressed genes (DEGs), heatmaps and clustering analysis

Those enrolled patients were divided into subtype A and B based on ORGS. The lists of ‘cell proliferation’ genes (GO:0008283) and ‘cell differentiation’ genes (GO:0030154) were downloaded from Gene Ontology (http://geneontology.org/). In supervised clustering, we selected the set of proliferation and differentiation genes (*n* = 312, and *n* = 1397) that differentially expressed between subtype A and B based on ORGS (*P* < 0.05), and heatmaps were plotted using java treeview [15]. In unsupervised clustering, hierarchical clustering was performed using cluster 3.0 [14]. Distances based on the uncentered correlation distance were calculated for input into an agglomerative algorithm and average linkage was selected for the clustering method.

## 3 Results

### 3.1 Development of gene signature predictive of recurrence‐free survival

To determine which genes influence patient survival, a gene signature was developed using the Cox proportional hazard model. These genes were evaluated for their effect on recurrence‐free survival in the TCGA training cohort. We selected 210 genes with *P*‐values < 0.005. Of these 210 genes, 123 were associated with an increased risk of recurrence (HR > 1), and 87 were associated with a decreased risk of recurrence (HR < 1). The 210 genes in the ORGS are listed in Table S1.

**Fig. 1:**
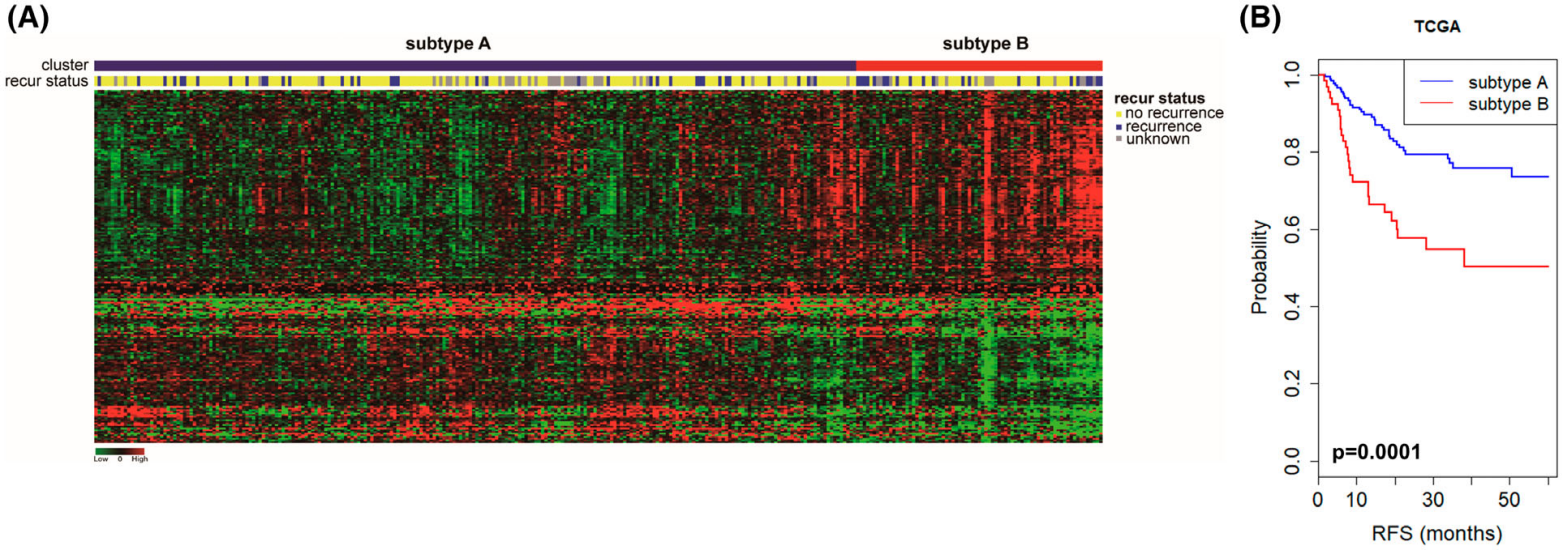
Development of a gene set correlated with recurrence‐free survival (RFS) in oral squamous cell carcinoma (OSCC). (A) Expression patterns of 210 genes related to recurrence in the cancer genome atlas (TCGA) cohort (*n* = 315). Green indicates low expression and red indicates high expression. In the recurrence (recur) panel, yellow ㅛ indicates patient without recurrence and blue indicates patient with recurrence. (B) Kaplan–Meier curve of the comparison between subtype a (*n* = 232) and subtype B (*n* = 83) in TCGA cohort. The significance was calculated by log‐rank test.

To identify patient subpopulations, hierarchical cluster analysis was performed using the ORGS as a predictor (Fig. 1A). Patients were classified as OSCC subtype A (*n* = 232) or OSCC subtype B (*n* = 83). Patients with subtype B OSCC had poorer prognosis and lower survival (Fig. 1B). In addition, subtype B samples showed an expression pattern in which the 123 genes associated with an increased risk of death were upregulated and the 87 genes associated with a decreased risk of death were downregulated.

### 3.2 Validation of the ORGS

**Fig. 2:**
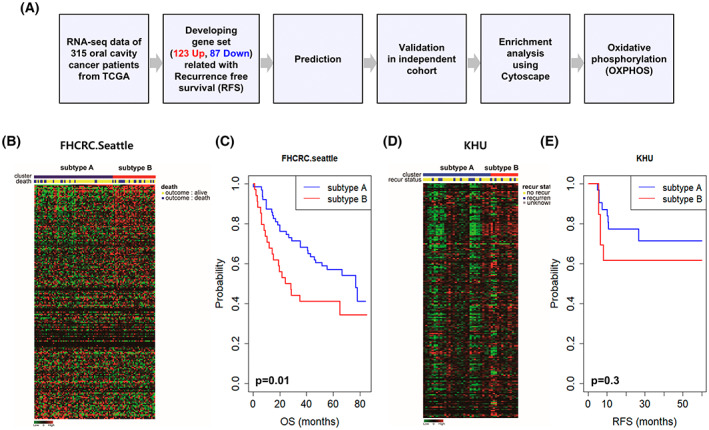
Validation of the OSCC recurrence‐related gene signature (ORGS) in an independent cohort. (A) Schematic diagram for developing the ORGS to predict recurrence in oral squamous cell carcinoma (OSCC). (B) Expression patterns of 210 genes with recurrence in the Fred Hutchinson Cancer Research Center (FHCRC) cohort (*n* = 87). (C) Kaplan–Meier curve of overall survival in the FHCRC cohort based on the OSCC subtype. (D) Heatmap analysis of ORGS in the Kyung Hee University (KHU) cohort (*n* = 45). (E) Kaplan–Meier curve of recurrence‐free survival in the KHU cohort based on the ORGS (*n* = 210). Significance of Kaplan–Meier curve was calculated by log‐rank test.

The ORGS was validated in the external FHCRC and KHU cohorts (Fig. 2A). In these independent cohorts, the ORGS was verified to have a significant prognostic value. In the FHCRC cohort, patients were also classified as having subtypes A or B according to the SVM predictor. Figure 2B shows a heatmap of the ORGS genes in the FHCRC cohort. Similar to the results from the TCGA cohort, patients with subtype B OSCC were more likely to have significantly shorter OS and poorer prognosis than patients with subtype A in the FHCRC dataset (Fig. 2C).

The ORGS was also validated in the KHU cohort using the method described above. Figure 2D shows the heatmap of ORGS genes using RNA‐seq data from the KHU cohort. Although not statistically significant, we did observe a trend that patients with subtype B had shorter overall survival (Fig. 2E).

### 3.3 The ORGS is an independent prognostic predictor in OSCC

To evaluate the effect of the ORGS on survival, univariate and multivariate Cox proportional hazard regression analyses were performed in the TCGA, FHCRC, and KHU cohorts (*n* = 457) based on the available clinical data. The combination of the ORGS and other clinicopathological factors (e.g., patient age, sex, smoking history, HPV infection, and cancer stage) was used for the Cox proportional hazard regression analysis. In the univariate analysis, ORGS subtype (subtype A vs. subtype B), smoking (smoker vs. non‐smoker), and cancer stage (I and II vs. III and IV) were significantly associated with OS. In the multivariate analysis, the ORGS subtype was the only significant factor (HR 1.512, 95% confidence interval 1.021–2.239; *P* = 0.039; Table 2). Therefore, this signature may be a promising independent prognostic predictor.

**Table 2:** Univariate and multivariate cox proportional hazard regression analysis of overall survival in the TCGA, FHCRC and KHU cohort (*n* = 457).

Variables	Univariate		Multivariate	
	HR (95% CI)	*P*‐value	HR (95% CI)	*P*‐value
ORGS (subtype B)	1.576 (1.066–2.331)	**0.0227**	1.512 (1.021–2.239)	**0.039**
Gender (male)	1.136 (0.761–1.697)	0.532	1.102 (0.721–1.686)	0.651
Age (> 60 years)	1.296 (0.895–1.877)	0.169	1.377 (0.942–2.013)	0.0989
Smoking (Yes)	1.561 (1.002–2.433)	**0.0492**	1.476 (0.928–2.348)	0.0997
HPV status (HPV‐positive)	0.713 (0.291–1.748)	0.46	0.634 (0.257–1.563)	0.3227
Stage (stage III & IV)	1.580 (1.014–2.460)	**0.043**	1.524 (0.972–2.391)	0.0665

Bold value indicates statistical significance by setting *P* < 0.05.

### 3.4 The ORGS is associated with OXPHOS

**Fig. 3:**
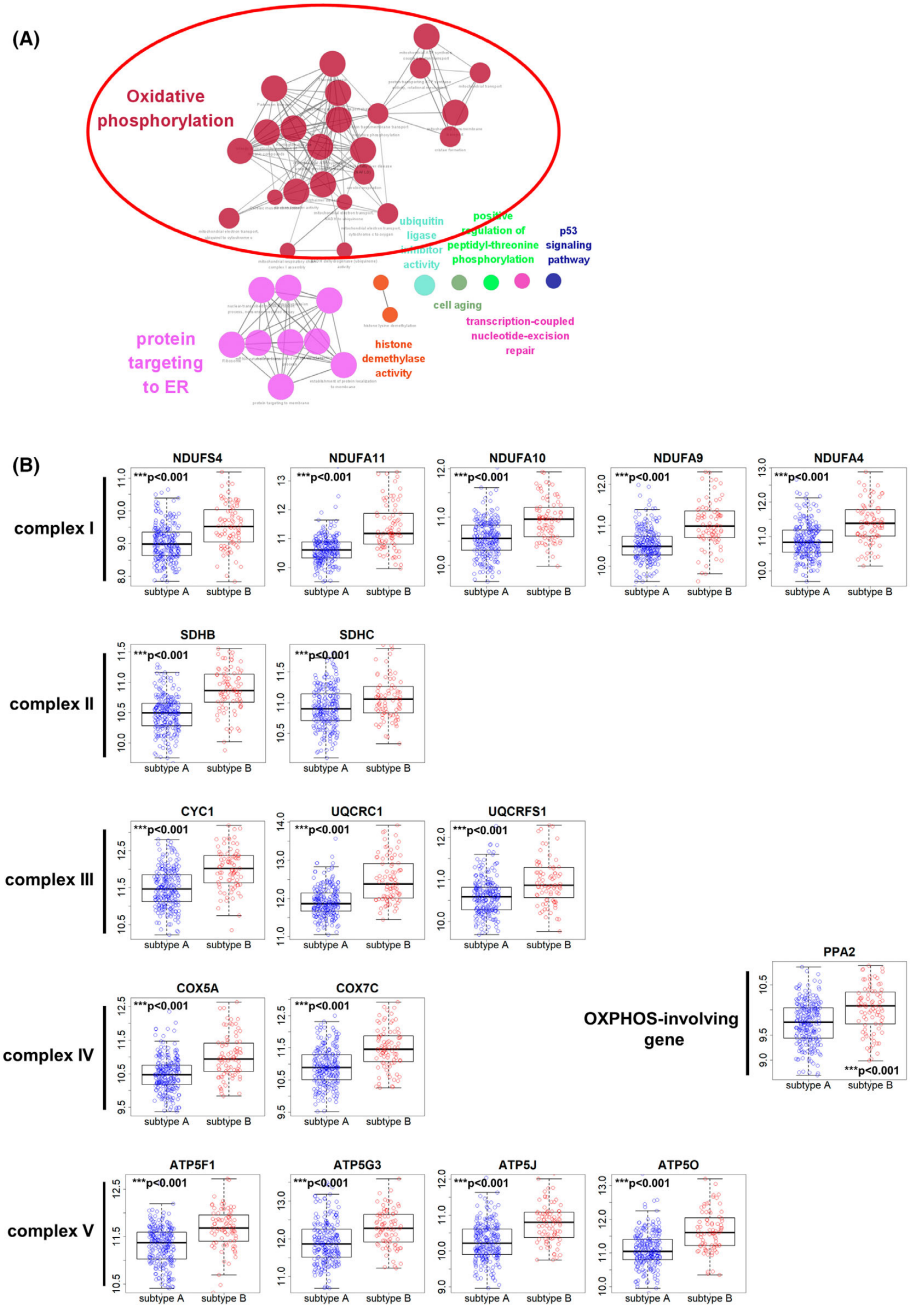
The OSCC recurrence‐related gene signature (ORGS) is associated with oxidative phosphorylation (OXPHOS). (A) Pathways related to the ORGS with *P* < 0.05. Pathway enrichment was conducted using the cluego application in cytoscape. (B) Expression of 17 OXPHOS genes in subtype a (*n* = 232) and subtype B (*n* = 83) oral squamous cell carcinoma (OSCC) in the cancer genome atlas (TCGA) cohort. OXPHOS genes were upregulated in subtype B (***P < 0.001). Error bars, SD. Significance was calculated by Welch's two sample *t*‐test.

To evaluate the importance of the ORGS in OSCC, an interaction network was generated using cytoscape, and pathway annotation analysis was performed using the plugin cluego. We also performed GO enrichment and KEGG pathway analyses of the 210 genes using cluego (Table S3). The GO biological processes associated with the ORGS included OXPHOS, respiratory ETC, protein targeting to the ER, nuclear‐transcribed mRNA catabolic process, and mitochondrial ATP synthesis coupled electron transport, among others. The KEGG pathway analysis indicated that the ORGS was associated with OXPHOS, Parkinson's disease, and thermogenesis. A summary of the genes involved in these processes is presented in Fig. 3A.

We decided to further explore the top‐ranked OXPHOS pathway in OSCC. Mitochondria function in cellular metabolism, producing the bulk of cellular ATP via OXPHOS. During this process, the ETC generates ATP using a proton gradient. In humans, the ETC comprises five multi‐subunit protein complexes: NADH dehydrogenase (complex I), succinate dehydrogenase (complex II), coenzyme Q:cytochrome c reductase (complex III), cytochrome c oxidase (complex IV), and ATP synthase (complex V) [21]. Seventeen of the genes in the ORGS were involved in OXPHOS: NDUFS4, NDUFA11, NDUFA10, NDUFA9, NDUFA4 (complex I), SDHB, SDHC (complex II), CYC1, UQCRC1, UQCRFS1 (complex III), COX5A, COX7C (complex IV), ATP5F1, ATP5G3, ATP5J, ATP5P (complex V), and PPA2 (OXPHOS‐involved gene). Interestingly, the mRNA expression levels of all 17 OXPHOS genes were significantly upregulated in subtype B OSCC compared to subtype A OSCC in the TCGA cohort (Fig. 3B).

### 3.5 MED30 is an upstream regulator of OXPHOS genes

Identifying the upstream regulator of OXPHOS can provide insight into understanding the underlying biology by which this potential mechanism gets activated in OSCC. To identify the upstream regulators of OXPHOS genes, we performed IPA upstream regulator analysis.

The eight transcriptional regulators predicted to be upstream regulators of OXPHOS genes are presented in Table S4. A Kaplan–Meier estimator was used for the survival analysis of these eight transcriptional regulators (Fig. S1A). Patients with high MED30 expression had significantly shorter OS than those with low MED30 expression. This suggested that the transcription factor MED30 may be more active in subtype B than in subtype A. We confirmed that MED30 was increased in subtype B compared to subtype A in the TCGA cohort (Fig. S1B). Therefore, MED30 is a promising candidate upstream regulator that regulates multiple genes that contribute to OXPHOS.

### 3.6 MED30 regulates OXPHOS genes at the transcriptional level by affecting histone acetylation

**Fig. 4:**
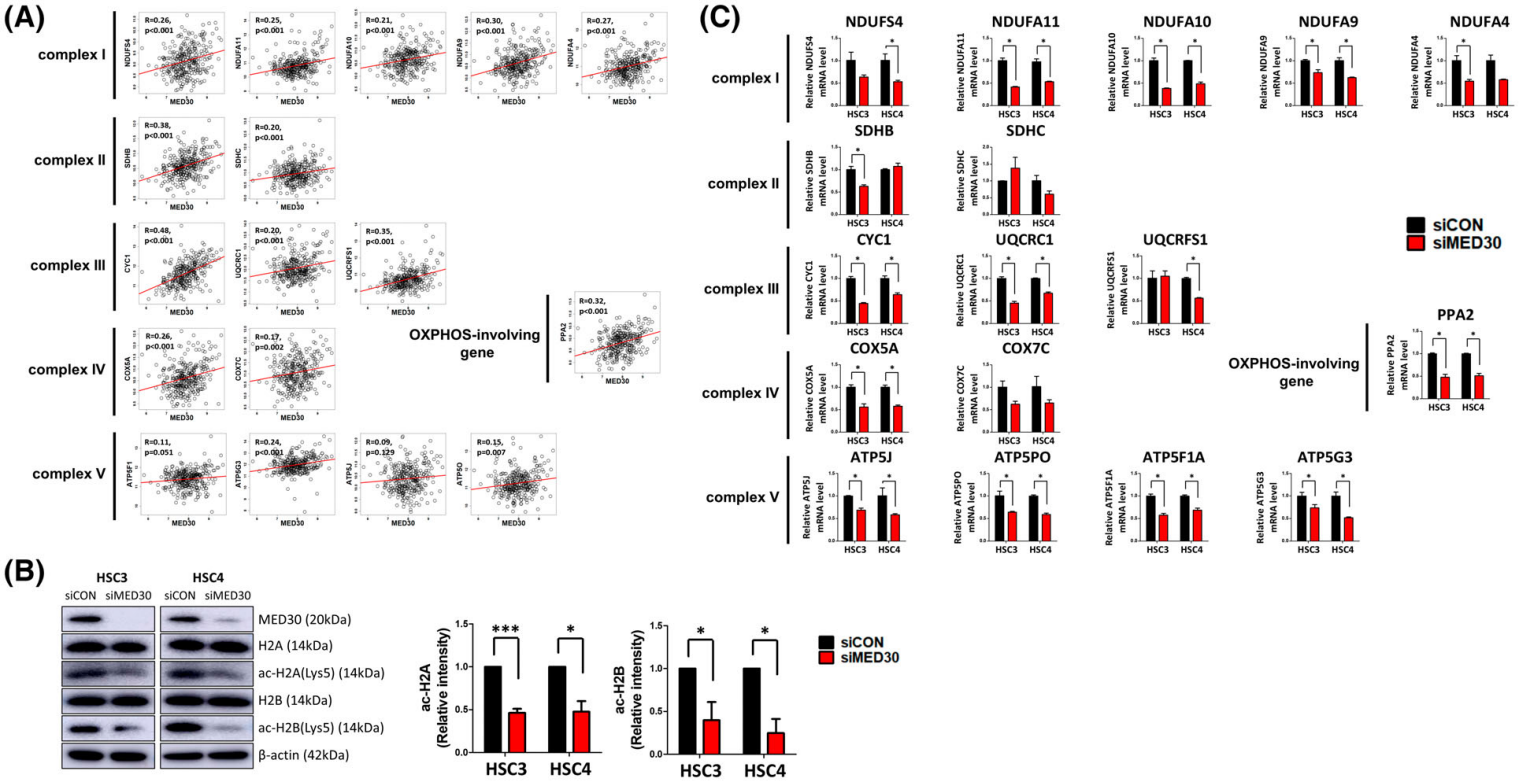
Mediator complex subunit 30 (MED30) regulates oxidative phosphorylation (OXPHOS) genes at the transcriptional level by regulating histone acetylation. (A) The correlation plot between MED30 and OXPHOS gene expression in TCGA cohort (*n* = 315). The correlation coefficient value was calculated using Pearson's correlation method. *R*; correlation coefficient value, *P*; *P*‐value. (B) Western blot of histones H2A, ac‐H2A, H2B and ac‐H2B in head and neck squamous cell carcinoma (HNSCC) cells 24 h after transfection of siMED30. Quantitative analysis of expression of ac‐H2A and ac‐H2B from three independent experiments. Error bars, SEM. *P < 0.05, ***P < 0.001. Significance was calculated by a two‐tailed *t*‐test. (C) qRT‐PCR of OXPHOS genes in siCON and siMED30 HNSCC cells. Expression was standardized to that of β‐Actin. Results are presented as relative gene expression (*n* = 3; *P < 0.05). Error bars, SEM. Significance was calculated by a two‐tailed *t*‐test.

Correlation analysis was performed on the expression of the MED30 and OXPHOS genes (Fig. 4A). The expression of OXPHOS genes positively correlated with the expression of MED30. In particular, NDUFA9, SDHB, CYC1, UQCRFS1, and PPA2 were strongly correlated with MED30 (*R* = 0.30, 0.38, 0.48, 0.35, and 0.32, respectively; all *P* < 0.001). COX7C, ATP5FS1, ATP5J, and ATP5PO showed weak correlations with MED30 (*R* = 0.17, *P* = 0.002; *R* = 0.11, *P* = 0.051; *R* = 0.09, *P* = 0.129; and *R* = 0.15, *P* = 0.007, respectively).

We next examined how MED30, a single factor, could regulate multiple OXPHOS genes. MED30 is a coactivator of RNA polymerase II and is involved in transcription [22]. Therefore, we hypothesized that MED30 regulates the expression of downstream genes by acetylating histones. To assess how MED30 affects histone acetylation, we transfected cells with siMED30. Western blotting demonstrated that siMED30 transfection diminished the levels of acetylated H2A and H2B (Fig. 4B). This result indicated that MED30 modulates histone acetylation, thereby inducing the transcription of downstream OXPHOS genes.

To better understand the expression patterns of OXPHOS genes and MED30, we transfected HSC3 and HSC4 cells with siRNA to silence MED30 (Fig. S2), and the expressions of OXPHOS genes and *MED30* were analyzed using qRT‐PCR (Fig. 4C). We found strong correlations between transcriptomic and qRT‐PCR results (Table S5). We observed a reduction in complex I, III, and IV genes in the cells transfected with siMED30. However, complex II and V genes were not reduced after MED30 knockdown, which contrasted with the transcriptomic data. The contradiction between the transcriptomic data and the qRT‐PCR data, especially in complex II and V genes, might be due to differences between a single cell line experiment and whole genome sequencing of heterogeneous cell types. However, our qRT‐PCR data confirm that MED30 regulates OXPHOS genes, especially those in complexes I, III, and IV.

### 3.7 MED30 influences MMP

Our qRT‐PCR analysis showed that MED30 specifically regulates genes belonging to ETC complexes I, III, and IV. The MMP generated by proton pumps (complexes I, III, and IV) is essential in the process of energy storage during OXPHOS [23]. Therefore, we examined whether knockdown of MED30 induced changes in the MMP.

**Fig. 5:**
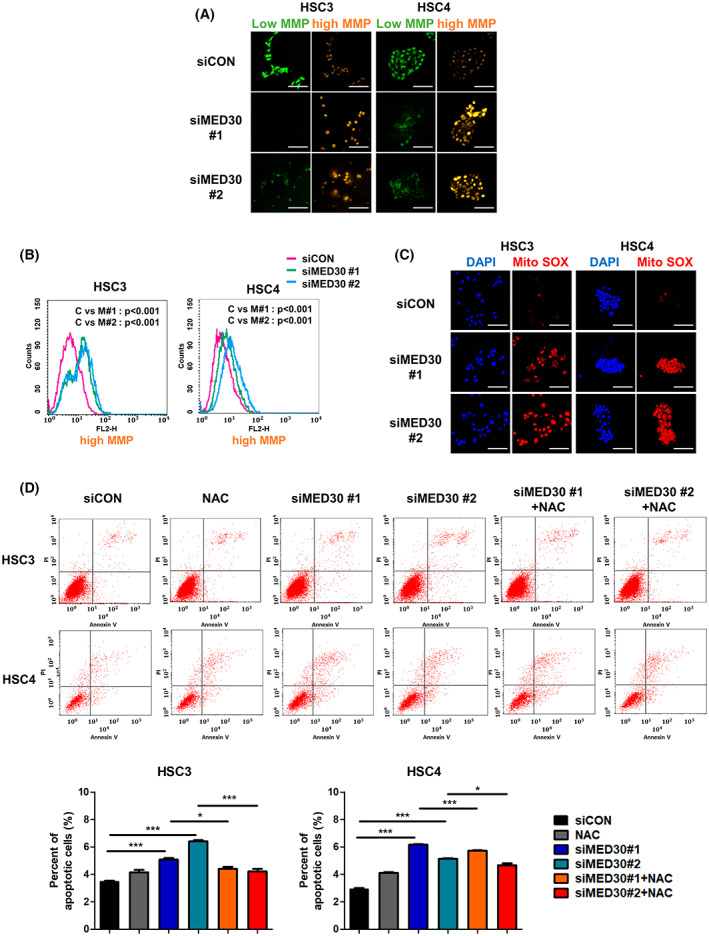
Mediator complex subunit 30 (MED30) knockdown regulates mitochondrial membrane potential (MMP) and increases reactive oxygen species (ROS). (A) Representative confocal images of HSC3 and HSC4 cells showing an increase in MITO‐ID fluorescence 24 h after transfection of siMED30. Scale bar = 100 μm. (B) Representative histograms of flow cytometry experiments demonstrating an increase in mean fluorescence intensity of MITO‐ID following siMED30 transfection as indicated. Significance was calculated by two‐sample Kolmogorov–Smirnov test. (C) Representative confocal images of HSC3 and HSC4 cells showing an increase in MitoSOX fluorescence 24 h after transfection of siMED30. Scale bar = 100 μm. (D) Apoptosis was detected by flow cytometry using annexin V‐FITC/PI double staining. 2 mm of *N*‐acetyl‐cysteine (NAC) was treated 24 h after transfection of siMED30. Quantitative analysis of apoptotic cells from three independent experiments. Error bars, SEM. *P < 0.05, **P < 0.01, ***P < 0.001. All experiments were performed in triplicates.

First, we evaluated the effect of MED30 knockdown on MMP in the two OSCC cell lines. We stained the cells with MITO‐ID, which stains mitochondria with high MMP orange and those with low MMP green. Knockdown of MED30 resulted in the accumulation of orange fluorescence, indicating that loss of MED30 results in high MMP (Fig. 5A). The FL‐2 channel detects orange fluorescence caused by dye aggregation in mitochondria with an intact membrane potential, implying an increase in energized mitochondria. Our data suggest an increase in energized mitochondria in siMED30 cells compared to control cells, as shown by the FL‐2 channel (Fig. 5B). MITO‐ID analysis of MMP in OSCC cells demonstrated that mitochondrial membrane depolarization was reduced in siMED30 cells compared to that in siCON cells.

### 3.8 MED30 knockdown increases ROS generation and induces apoptosis

Increased mitochondrial polarization is associated with greater ROS generation. Mitochondrial ROS (mtROS) generation in MED30 knockdown cells was evaluated using MitoSOX staining. As shown in Fig. 5C, mtROS levels were significantly increased in siMED30 cells compared to those in siCON cells.

Annexin V‐FITC/PI dual‐staining detection was used to evaluate the difference in apoptosis rates between siCON and siMED30 cells. siMED30 transfection considerably increased apoptosis compared with siCON transfection (Fig. S3). Further, OSCC cells were subjected to apoptotic cell death upon transfection of siMED30, but the treatment of 2 mm *N*‐acetyl‐cysteine (NAC) suppressed this process (Fig. 5D). These data suggest that MED30 knockdown may induce apoptosis via ROS generation.

## 4 Discussion

Accurate prediction of survival is important for counseling, treatment planning, follow‐up, and postoperative risk assessment in patients with OSCC. Therefore, there has been increased interest in the development of prognostic predictors [24]. Previous studies have identified gene‐based prognostic signatures and survival‐related alternative splicing genes as prognostic predictors of OSCC [25, 26, 27]. However, these gene signatures have not been used in clinical practice, and recurrence‐related biological pathways in OSCC remain poorly explored, necessitating the development of useful and accurate prognostic predictors for OSCC [28]. Here, we employed a bioinformatics approach using OSCC data extracted from the TCGA HNSCC database and created the ORGS. The training, validation, and external independent cohorts proved that the ORGS was robust and had a high prediction ability. GO enrichment and KEGG pathway analyses revealed that OXPHOS is a potential pathway involved in this signature.

OXPHOS has emerged as a novel target for cancer therapy. A previous study demonstrated that the subpopulation of dormant tumor cells surviving oncogene ablation, which are responsible for tumor relapse, has features of CSCs and relies on OXPHOS for survival in pancreatic cancer [29]. These surviving cells show high sensitivity to OXPHOS inhibitors, which can block tumor recurrence. Furthermore, marizomib, a dual inhibitor of the proteasome and OXPHOS, reduces lung and brain metastases in patients with triple‐negative breast cancer [30]. In AML, cytarabine‐induced chemo‐resistant cells display high levels of ROS and retain active polarized mitochondria, consistent with a high OXPHOS status. An energetic shift toward a low OXPHOS status with cytarabine treatment enhances its anti‐leukemic effects in AML. These results suggest that targeting OXPHOS is a promising avenue for designing new therapeutic strategies for AML [31]. Our findings suggest that increased OXPHOS is associated with poor prognosis in patients with OSCC. To systematically explore how changes in specific biological pathways translate into different prognoses in patients, we performed IPA upstream regulator analysis to identify upstream regulators of OXPHOS genes. Eight candidate genes were ranked as upstream regulators. We explored MED30 as a key regulator of OXPHOS. This mediator is a multi‐subunit complex that plays a central role in the regulation of gene transcription. Previous studies have described the expression and role of mediator complex subunits in carcinogenesis, progression, and invasion. However, MED30 has only been examined in gastric, renal, and breast cancers [32, 33, 34]. In gastric cancer, overexpression of MED30 increases proliferation, migration, and invasion. In addition, overexpression of MED30 in papillary renal cell carcinoma is significantly associated with poorer overall survival. In clear cell renal cell carcinoma, the knockdown of MED30 results in a significant decrease in proliferation, migration, and invasion. However, the specific role of MED30 in tumorigenesis remains poorly characterized.

Intriguingly, Kaplan–Meier analysis showed that patients with high MED30 had poor recurrence‐free survival (RFS, Fig. S4A). This result showed the potential that up‐regulation of MED30 has significant pathological implications in OSCC. To investigate the functional role of MED30 in OSCC metastasis, we used siRNA transfected OSCC cells. Transwell invasion assay revealed that MED30 knockdown in HSC3 and HSC4 cells reduced cell invasion compared with siCON transfected cells (Fig. S4B). Collectively, this result indicated that MED30 could stimulate the aggressive and metastatic phenotype of OSCC. MED30 is important for transcription, including the organization of chromatin architecture and the regulation of RNA polymerase II pre‐initiation, initiation, re‐initiation, pausing, and elongation [35]. It interacts with other transcriptional regulators, such as activators, co‐activators, and general transcription factors, to modify histones, which regulates the expression of target genes [36]. Upon transduction of appropriate signals via mediator recruitment, RNA polymerase II is redirected to synthesize full‐length transcripts [37]. The question remained as to whether MED30 serves as the main regulator of histone modification. In this study, we observed an association between MED30 and histone acetylation. It is possible that MED30 plays an important role in the regulation of downstream genes through histone acetylation.

Histone acetylation is regulated by histone lysine acetyltransferases and is associated with ‘open’ chromatin structures. Histone acetylation at gene promoter or enhancer regions helps recruit transcription factors, leading to the upregulation of gene expression [38]. Consistent with this, Chen et al. [39] showed that enhancement of histone acetylation consequently increased the expression of OXPHOS gene in prostate cancer cells.

**Fig. 6:**
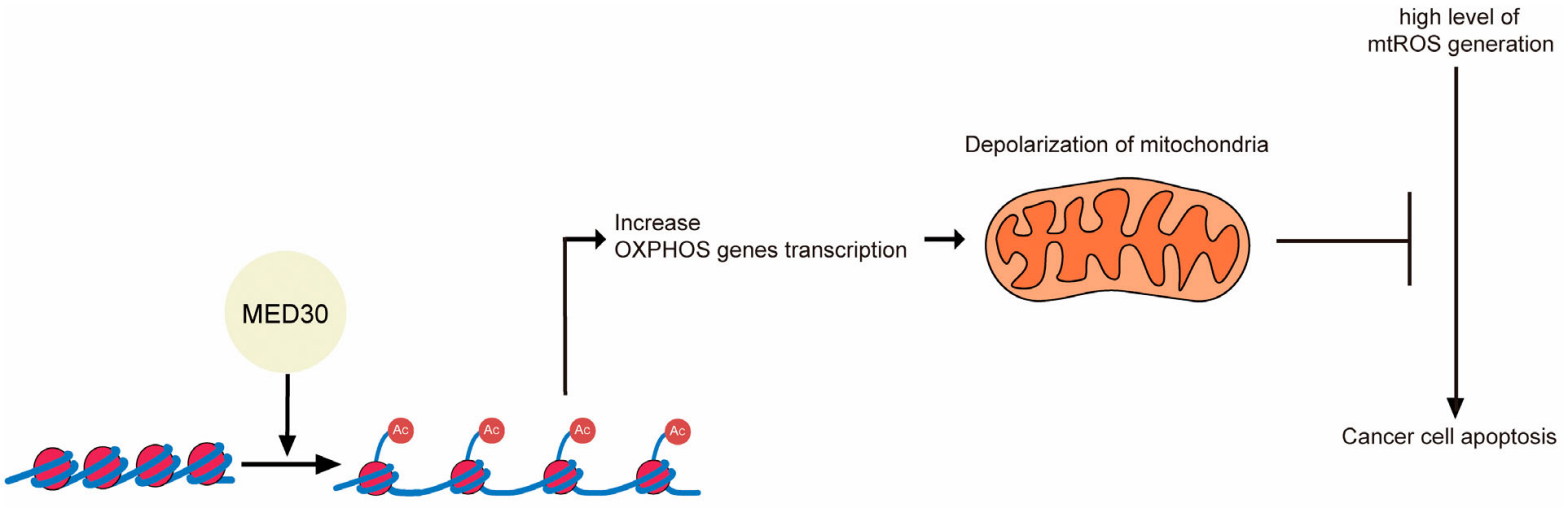
Mediator complex subunit 30 (MED30) may have a role in acetylating histone proteins and increasing oxidative phosphorylation (OXPHOS) gene transcription. In cancer cells, mitochondrial reactive oxygen species (ROS) generation may increase while mitochondrial membrane potential (MMP) is maintained at a high level, which may lead to cancer cell apoptosis. However, increased OXPHOS gene transcription by MED30 promotes depolarization of mitochondria, which may inhibit mitochondrial ROS (mtROS) generation and cancer cell apoptosis.

This study revealed the specific mechanism of action of MED30 in OSCC. Our data suggest that MED30 modulates histone modification and regulates the expression of OXPHOS genes (Fig. 6). These results suggest that MED30 regulates OXPHOS genes by regulating histone acetylation, leading to poor prognosis in OSCC patients.

Both supervised and unsupervised clustering were used as the validation method of ORGS. In supervised clustering, subtypes A and B patients based on ORGS were enrolled. In our results, subtype B showed poor prognosis than subtype A in OSCC patients (Fig. 1B). Heatmaps were plotted with proliferation and differentiation genes differently expressed between subtypes A and B. This result indicates that the expression patterns of proliferation and differentiation genes in subtypes A and B are different (Fig. S5A,B). Furthermore, heatmaps drawn using unsupervised clustering (here hierarchical clustering) also show differences in the expression of proliferation and differentiation genes between the two subtypes (Fig. S5C,D). These results suggest that ORGS can also predict proliferative and differentiative cell conditions in OSCC patients.

Previous studies have been analyzed to elucidate the relationship between OXPHOS and clinical outcomes in OSCC patients. Frederick et al. [40] showed that the expression of OXPHOS genes and the survival of patients with oral cavity squamous cell carcinoma (OCSCC) are not explained by a simple linear relationship. In addition, various factors such as mitochondrial copy number, tumor purity and immunocyte infiltration were considered for mitochondrial activity correlated with clinical outcome. Therefore, they emphasized that multiple prospectives should be considered in targeting mitochondrial activity. On the other hand, these results not only reveal that OXPHOS may act as a key pathway leading to poor prognosis in patients but also elucidates the sequence of MED30‐mediated OXPHOS regulation.

In this study, we highlight the remarkable mechanism of MED30‐mediated OXPHOS, which regulates MMP and ROS generation. MED30 significantly regulated genes belonging to ETC complexes I, III, and IV, which are proton pumps involved in MMP regulation. Our mtROS and apoptosis results showed that knockdown of MED30 induced mitochondrial polarization and apoptosis. Reduction of OXPHOS gene expression by knockdown of MED30 allows MMP to be maintained at a high level. Even without mitochondria impairment, these higher MMP causes a high level of mtROS generation, which increases apoptosis [41]. The concept that a higher (more polarized) MMP is associated with greater mtROS generation is thought to be due to slowed electron transport [42].

## 5 Conclusions

We developed the ORGS, which consists of genes highly correlated with OSCC recurrence. Additionally, we demonstrated the potential of OXPHOS, which was highly associated with the ORGS, as a key hallmark of malignancy in OSCC. Our data also showed that this malignant mechanism was dependent on high expression of MED30, which affected histone acetylation, increased expression of OXPHOS genes, and consequently elevated OXPHOS status.

## Conflict of interest

The authors declare no conflict of interest.

## Author contributions

Conceptualization: Y‐GE; Data curation: JKN, SRW, and YCL; Formal analysis: JKN; Methodology: SRW, MKL, S‐GK, and Y‐GE; Project administration: SRW, and Y‐GE; Supervision: Y‐GE; Validation: JKN, JWL, YCL, and S‐GK; Visualization: MKL, and JWL; Roles/Writing – original draft: JKN; Writing – review & editing: Y‐GE.

## Supporting information


**Fig. S1.** Candidate for upstream regulator of OXPHOS genes. MED30 was the most predicted suitable upstream regulators.

**Fig. S2.** MED30 is a transcription regulator of OXPHOS genes.

**Fig. S3.** Inhibition of MED30 induces apoptosis in cancer cells.

**Fig. S4.** MED30 expression is related to patient's survival and regulate invasion ability in OSCC.

**Fig. S5.** ORGS can also predict proliferation and differentiation related genes.

**Table S1.** ORGS genes and OXPHOS related genes are listed.

**Table S2.** Oligonucleotide PCR primers based on the *Homo sapiens* genome.

**Table S3.** GO and KEGG pathway lists related to ORGS.

**Table S4.** The list of upstream regulator of OXPHOS genes from IPA analysis.

**Table S5.**Correlation coefficient value of MED30 and OXPHOS genes. OXPHOS genes mRNA expression was analyzed to determine MED30 regulates OXPHOS genes in transcriptional level.

## Data Availability

Raw data for RNA‐seq and microarray data are available in the public open access repository (NCBI database). All data analyzed during this study are included in this manuscript.
