# Supplementary figures and images for: Gene signature predicting recurrence in oral squamous cell carcinoma is characterized by increased oxidative phosphorylation
Source: Mol Oncol. 2022 Nov 23;17(1):134–49. doi: 10.1002/1878-0261.13328 (PMC9812830; doi:10.1002/1878-0261.13328)

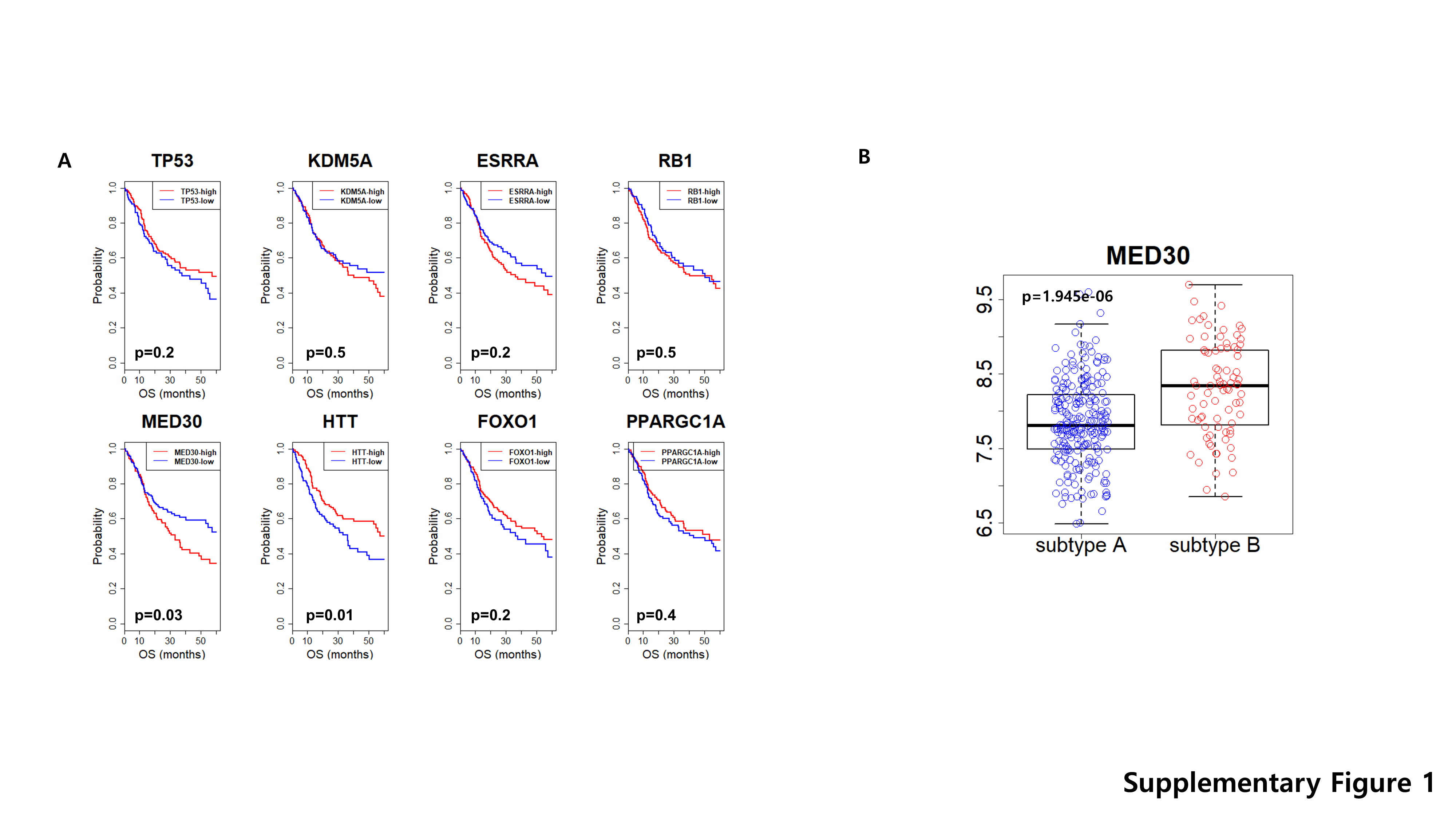

Supplement: Supplementary file 1 — Fig. S1. Candidate for upstream regulator of OXPHOS genes. MED30 was the most predicted suitable upstream regulators. [file MOL2-17-134-s007.TIF]

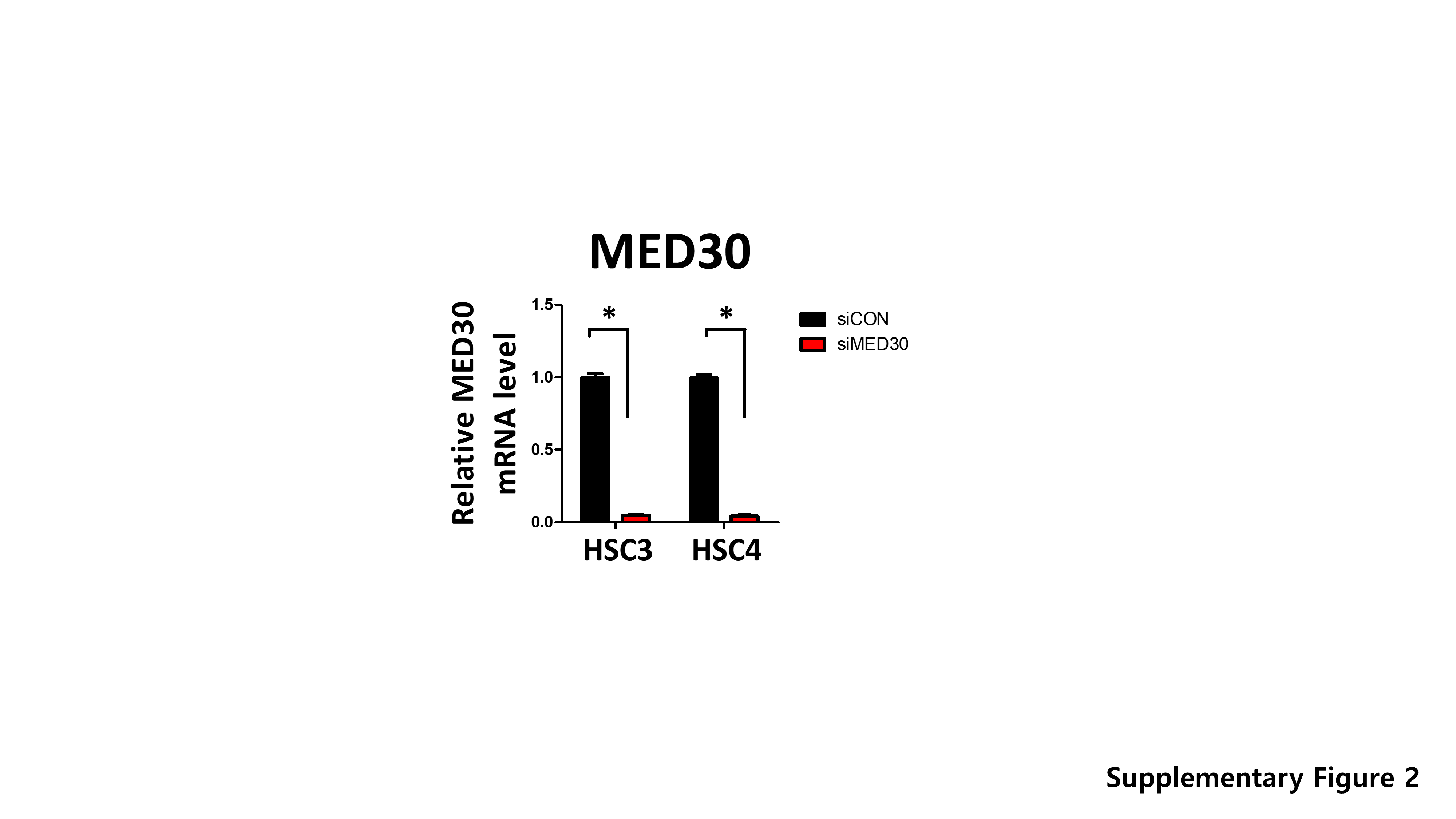

Supplement: Supplementary file 2 — Fig. S2. MED30 is a transcription regulator of OXPHOS genes. [file MOL2-17-134-s005.TIF]

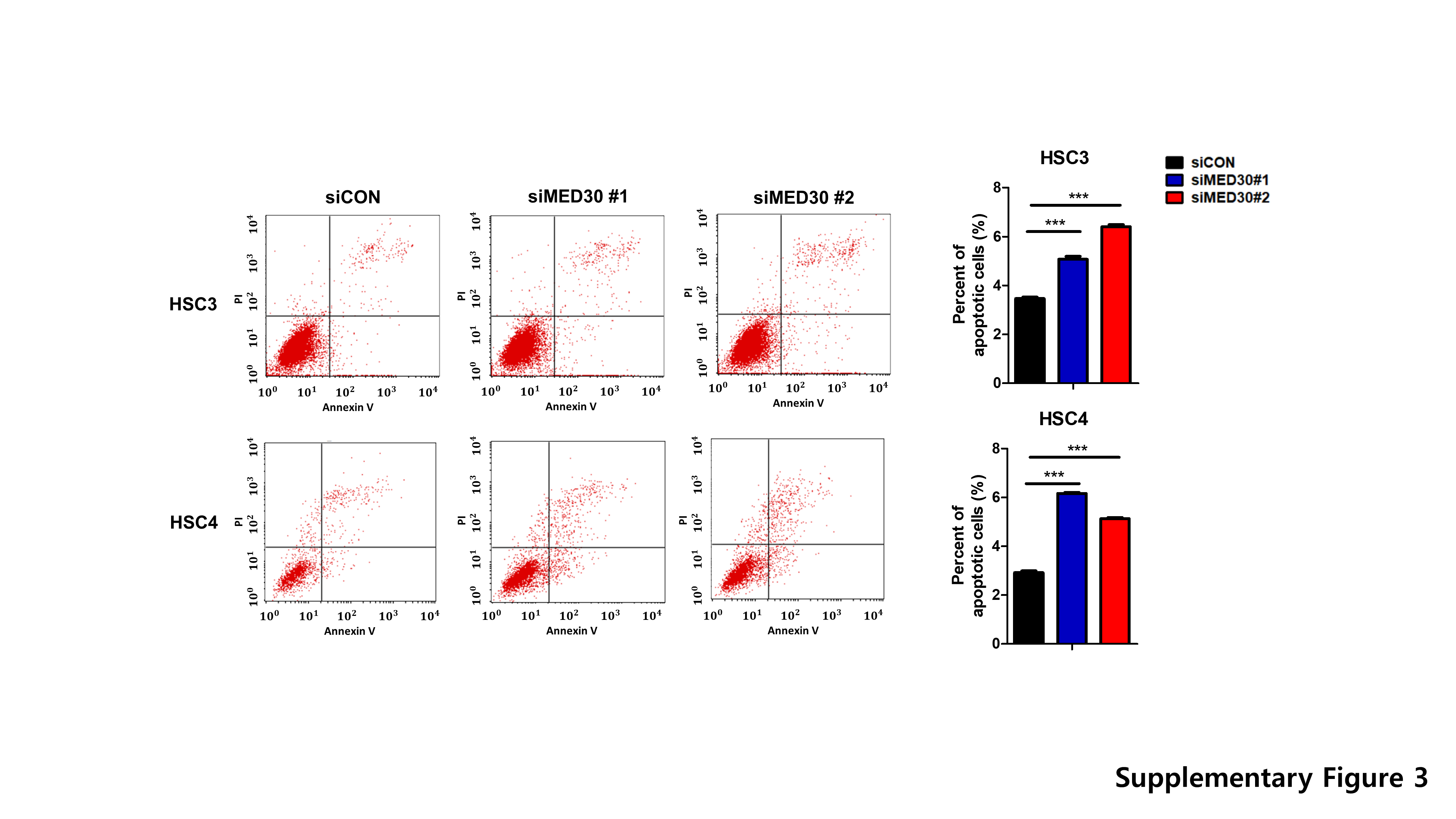

Supplement: Supplementary file 3 — Fig. S3. Inhibition of MED30 induces apoptosis in cancer cells. [file MOL2-17-134-s006.tif]

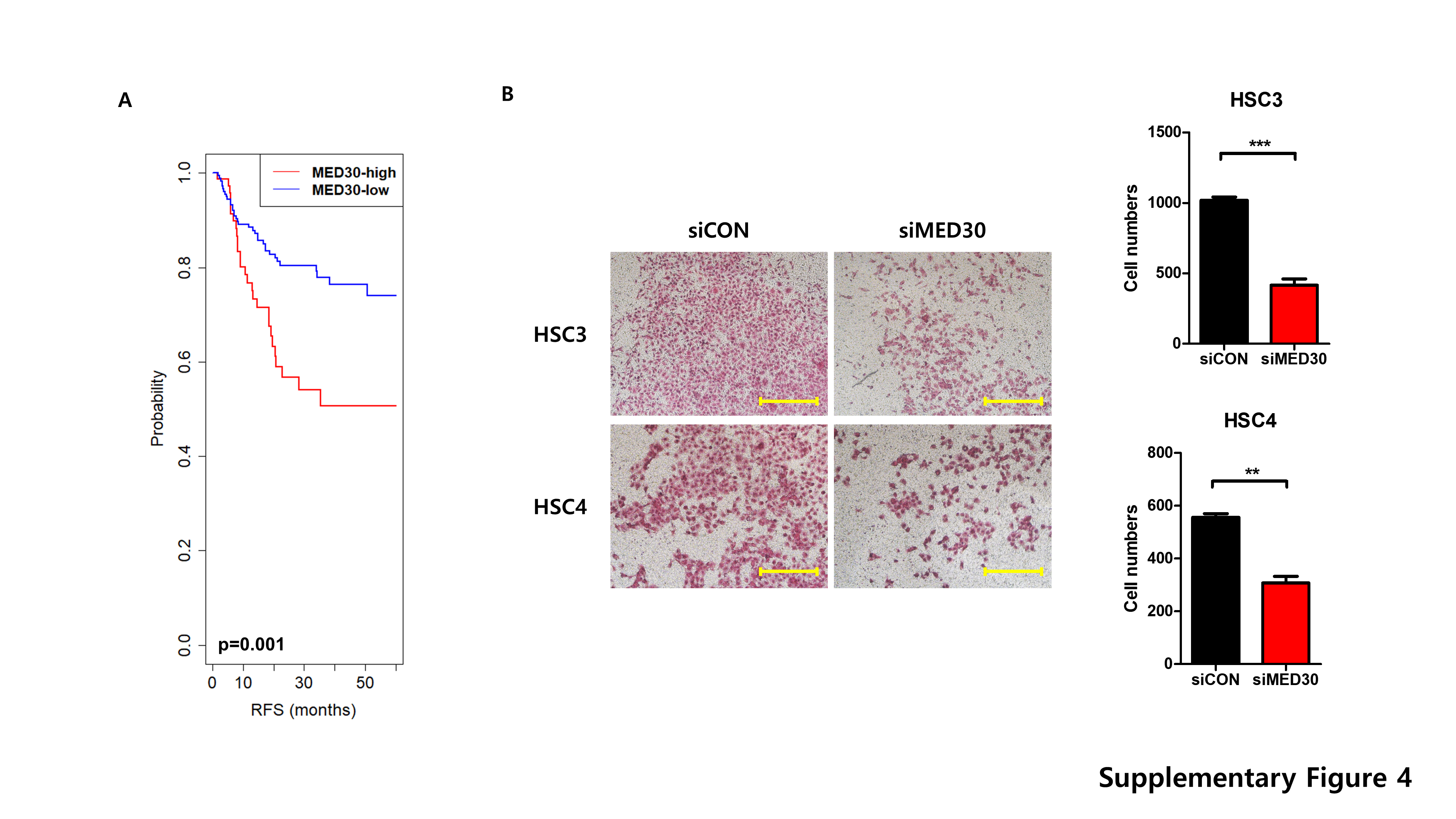

Supplement: Supplementary file 4 — Fig. S4. MED30 expression is related to patient's survival and regulate invasion ability in OSCC. [file MOL2-17-134-s004.TIF]

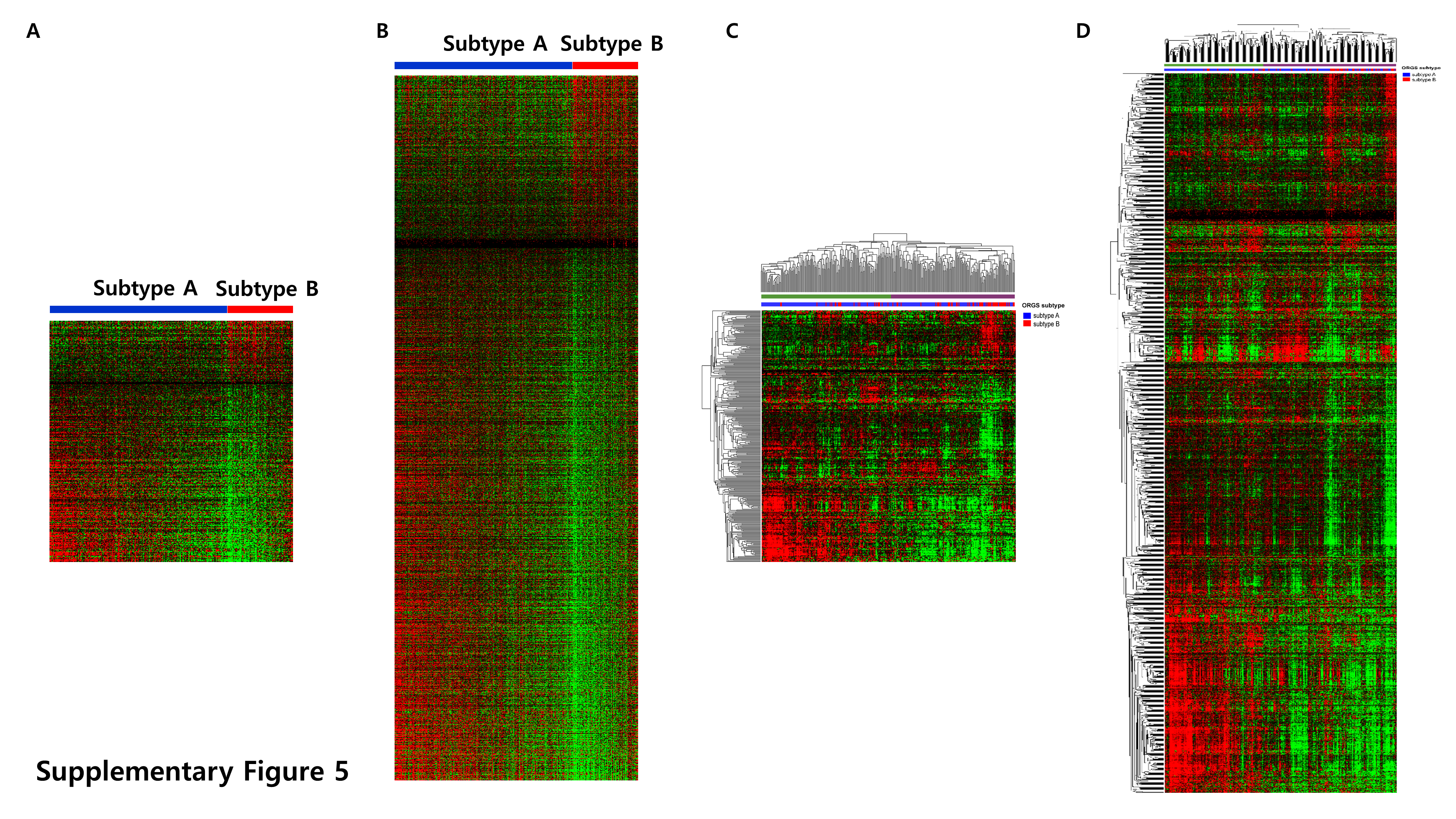

Supplement: Supplementary file 5 — Fig. S5. ORGS can also predict proliferation and differentiation related genes. [file MOL2-17-134-s009.TIF]
