# Supplementary material for: Gene signature predicting recurrence in oral squamous cell carcinoma is characterized by increased oxidative phosphorylation
Source: Mol Oncol. 2022 Nov 23;17(1):134–49. doi: 10.1002/1878-0261.13328 (PMC9812830; doi:10.1002/1878-0261.13328)
Supplement: Supplementary file 7 — Table S2. Oligonucleotide PCR primers based on the Homo sapiens genome. [file MOL2-17-134-s010.docx]

**Supplementary Table2**. Oligonucleotide PCR primers based on the *Homo sapiens* genome.

| **Gene** | **Accession num.** | **Primer sequence** | **Orientation** |
| --- | --- | --- | --- |
| NDUFS4 | [**NM_002495.4**](https://www.ncbi.nlm.nih.gov/nuccore/NM_002495.4) | GACCAGACTCAAGACACACAA | Forward |
|  |  | ACCATGTTGGATAAGGGATCAG | Reverse |
| NDUFA11 | [**NM_175614.4**](https://www.ncbi.nlm.nih.gov/nuccore/NM_175614.5) | TCACACTCAATCCTCCGGGCACCTT | Forward |
|  |  | TGATGCAGGTGGTGAGGCCAAACA | Reverse |
| NDUFA10 | [**NM_004544.4**](https://www.ncbi.nlm.nih.gov/nuccore/NM_004544.4) | GAGCTCATCAGACTGACCGT | Forward |
|  |  | GTGTTGTACCCAGGGCTGTA | Reverse |
| NDUFA9 | [**NM_005002.5**](https://www.ncbi.nlm.nih.gov/nuccore/NM_005002.5) | GACCCAGGGTTGGTCATTGT | Forward |
|  |  | TCTCTGTAAGGAGACGGGCA | Reverse |
| NDUFA4 | [**NM_002489.4**](https://www.ncbi.nlm.nih.gov/nuccore/NM_002489.4) | TCCAGATGTTTGTTGGGACA | Forward |
|  |  | CTGGACGTTCCTTCTTCAGC | Reverse |
| SDHB | [**NM_003000.2**](https://www.ncbi.nlm.nih.gov/nuccore/NM_003000.3) | TTCCGAAGATCATGCAGAGAAG | Forward |
|  |  | GGAGTCAATCATCCAGCGATAG | Reverse |
| SDHC | [**NM_003001.3**](https://www.ncbi.nlm.nih.gov/nuccore/NM_003001.5) | CTTTAGCCCTCAGCTCTGTATC | Forward |
|  |  | TGCAGCCACCTCATCTTTAG | Reverse |
| CYC1 | [**NM_001916.5**](https://www.ncbi.nlm.nih.gov/nuccore/NM_001916.5) | CTCCCTGCTCACGGGCTACT | Forward |
|  |  | GGGTGCCATCGTCAAACTCTAA | Reverse |
| UQCRC1 | [**NM_003365.3**](https://www.ncbi.nlm.nih.gov/nuccore/NM_003365.3) | GGCCCAGTGAGAATGTCAGG | Forward |
|  |  | CGAGGGGCCTTGTAATGTGT | Reverse |
| UQCRFS1 | [**NM_006003.3**](https://www.ncbi.nlm.nih.gov/nuccore/NM_006003.3) | CCGATATTCCAGAAGGCAAGAA | Forward |
|  |  | GCAGACCAGACGTGAAGATAAA | Reverse |
| COX5A | [**NM_004255.4**](https://www.ncbi.nlm.nih.gov/nuccore/NM_004255.4) | TGGCTATCCAGTCAGTTCGC | Forward |
|  |  | TGTTACCCAGCGAGCATCAA | Reverse |
| COX7C | [**NM_001867.3**](https://www.ncbi.nlm.nih.gov/nuccore/NM_001867.3) | GCCATTTCATCTGTCCTCATTC | Forward |
|  |  | GGCATTAAGCATTCACGTCATA | Reverse |
| ATP5J | [**NM_001302213.1**](https://www.ncbi.nlm.nih.gov/nuccore/NM_001302213.1) | GTATCAGCAAGAGCTGGAGAG | Forward |
|  |  | GAACACACTCAACATCACCAAATA | Reverse |
| ATP5PO | [**NM_001697.3**](https://www.ncbi.nlm.nih.gov/nuccore/NM_001697.3) | ACTCGGGTTTGACCTACAGC | Forward |
|  |  | GCCTCACAAGCTTGGCAAAT | Reverse |
| ATP5F1A | [**NM_001001935.3**](https://www.ncbi.nlm.nih.gov/nuccore/NM_001001935.3) | TCTCAGTCTACGCCGCACTT | Forward |
|  |  | GACATCTCAGCAGTCCCACA | Reverse |
| ATP5G3 | [**NM_001301721.1**](https://www.ncbi.nlm.nih.gov/nuccore/NM_001301721.1) | CCCAGTAGGGACCCATTCAT | Forward |
|  |  | TAAGGTCAAGTGCCCTCCAG | Reverse |
| PPA2 | [**NM_176869.3**](https://www.ncbi.nlm.nih.gov/nuccore/NM_176869.3) | AAGCTACGCTATGTGGCGAAT | Forward |
|  |  | TTCATGGGGATCTTCCCAAGTC | Reverse |
| MED30 | [**NM_080651.4**](https://www.ncbi.nlm.nih.gov/nuccore/NM_080651.4) | CCCACCTCGTTTTGCTAGTGA | Forward |
|  |  | TCAGCTGTTGATTCTTCTGTTTGAG | Reverse |
| β-actin | [**NM_001101.5**](https://www.ncbi.nlm.nih.gov/nuccore/NM_001101.5) | GGACTTCGAGCAAGAGATGG | Forward |
|  |  | AGCACTGTGTTGGCGTACAG | Reverse |
