# Supplementary material for: Gene signature predicting recurrence in oral squamous cell carcinoma is characterized by increased oxidative phosphorylation
Source: Mol Oncol. 2022 Nov 23;17(1):134–49. doi: 10.1002/1878-0261.13328 (PMC9812830; doi:10.1002/1878-0261.13328)
Supplement: Supplementary file 9 — Table S4. The list of upstream regulator of OXPHOS genes from IPA analysis. [file MOL2-17-134-s001.docx]

**Supplementary table 4**. The list of upstream regulator of OXPHOS genes from IPA analysis.

| **Upstream Regulator** | **p-value** |
| --- | --- |
| KDM5A | 2.09E-14 |
| Esrra | 8.77E-13 |
| RB1 | 1.54E-10 |
| MED30 | 3.31E-07 |
| TP53 | 0.0000236 |
| HTT | 0.000182 |
| FOXO1 | 0.000271 |
| PPARGC1A | 0.000936 |
