# Supplementary material for: Gene signature predicting recurrence in oral squamous cell carcinoma is characterized by increased oxidative phosphorylation
Source: Mol Oncol. 2022 Nov 23;17(1):134–49. doi: 10.1002/1878-0261.13328 (PMC9812830; doi:10.1002/1878-0261.13328)
Supplement: Supplementary file 10 — Table S5.Correlation coefficient value of MED30 and OXPHOS genes. OXPHOS genes mRNA expression was analyzed to determine MED30 regulates OXPHOS genes in transcriptional level. [file MOL2-17-134-s002.docx]

**Supplementary Table 5.** Correlation coefficient alue of MED30 and OXPHOS genes. OXPHOS genes mRNA expression was analyzed to determine MED30 regulates OXPHOS genes in transcriptional level.

|  |  | **MED30** | | | | | | |
| --- | --- | --- | --- | --- | --- | --- | --- | --- |
|  |  | **TCGA data** | | **RT-PCR data** | | | | |
|  |  |  |  | HSC3 | | HSC4 | | |
| **OXPHOS** | **Gene** | Correlation r | p-value | Fold change  (siMED30  /siCON) | p-value | | Fold change  (siMED30  /siCON) | p-value |
| **Complex I** | **NDUFS4** | 0.258 | **<0.001** | 0.640 | 0.063 | | 0.535 | **0.026** |
|  | **NDUFA11** | 0.251 | **<0.001** | 0.417 | **0.002** | | 0.531 | **0.005** |
|  | **NDUFA10** | 0.211 | **<0.001** | 0.385 | **0.002** | | 0.490 | **0.001** |
|  | **NDUFA9** | 0.303 | **<0.001** | 0.733 | **0.010** | | 0.623 | **0.006** |
|  | **NDUFA4** | 0.273 | **<0.001** | 0.548 | **0.014** | | 0.576 | 0.126 |
| **Complex II** | **SDHB** | 0.378 | **<0.001** | 0.629 | **0.006** | | 1.073 | 0.368 |
|  | **SDHC** | 0.201 | **<0.001** | 1.384 | 0.167 | | 0.612 | 0.134 |
| **Complex III** | **CYC1** | 0.477 | **<0.001** | 0.445 | **<0.001** | | 0.639 | **0.001** |
|  | **UQCRC1** | 0.204 | **<0.001** | 0.453 | **<0.001** | | 0.674 | **0.001** |
|  | **UQCRFS1** | 0.354 | **<0.001** | 1.051 | 0.757 | | 0.561 | **0.008** |
| **Complex IV** | **COX5A** | 0.256 | **<0.001** | 0.561 | **0.006** | | 0.579 | **0.001** |
|  | **COX7C** | 0.174 | **0.002** | 0.622 | 0.120 | | 0.653 | 0.258 |
| **Complex V** | **ATP5F1A** | 0.110 | 0.051 | 0.578 | **0.010** | | 0.686 | **0.002** |
|  | **ATP5G3** | 0.237 | **<0.001** | 0.740 | **0.015** | | 0.517 | **0.008** |
|  | **ATP5J** | 0.086 | 0.129 | 0.689 | **0.045** | | 0.584 | **0.048** |
|  | **ATP5PO** | 0.152 | **0.007** | 0.643 | **0.027** | | 0.589 | **<0.001** |
| **OXPHOS-involving gene** | **PPA2** | 0.325 | **<0.001** | 0.477 | **0.003** | | 0.512 | **0.002** |
